# Response stopping under conflict: The integrative role of representational dynamics associated with the insular cortex

**DOI:** 10.1002/hbm.26643

**Published:** 2024-04-25

**Authors:** Filippo Ghin, Elena Eggert, Negin Gholamipourbarogh, Nasibeh Talebi, Christian Beste

**Affiliations:** ^1^ Cognitive Neurophysiology, Department of Child and Adolescent Psychiatry, Faculty of Medicine TU Dresden Dresden Germany

**Keywords:** action control, EEG, group ICA, insular cortex, MVPA, stopping, superior parietal cortex

## Abstract

Coping with distracting inputs during goal‐directed behavior is a common challenge, especially when stopping ongoing responses. The neural basis for this remains debated. Our study explores this using a conflict‐modulation Stop Signal task, integrating group independent component analysis (group‐ICA), multivariate pattern analysis (MVPA), and EEG source localization analysis. Consistent with previous findings, we show that stopping performance is better in congruent (nonconflicting) trials than in incongruent (conflicting) trials. Conflict effects in incongruent trials compromise stopping more due to the need for the reconfiguration of stimulus–response (S–R) mappings. These cognitive dynamics are reflected by four independent neural activity patterns (ICA), each coding representational content (MVPA). It is shown that each component was equally important in predicting behavioral outcomes. The data support an emerging idea that perception‐action integration in action‐stopping involves multiple independent neural activity patterns. One pattern relates to the precuneus (BA 7) and is involved in attention and early S–R processes. Of note, three other independent neural activity patterns were associated with the insular cortex (BA13) in distinct time windows. These patterns reflect a role in early attentional selection but also show the reiterated processing of representational content relevant for stopping in different S–R mapping contexts. Moreover, the insular cortex's role in automatic versus complex response selection in relation to stopping processes is shown. Overall, the insular cortex is depicted as a brain hub, crucial for response selection and cancellation across both straightforward (automatic) and complex (conditional) S–R mappings, providing a neural basis for general cognitive accounts on action control.

## Graphical abstract

The insular cortex is depicted as a brain hub, crucial for the recurrent handling of response‐representations during response selection and cancellation across both straightforward (automatic) and complex (conditional) stimulus–response mappings, providing a neural basis for general cognitive accounts on action control.

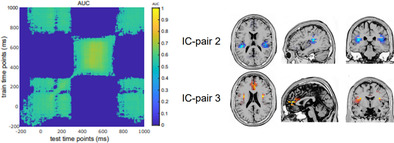

## 1 INTRODUCTION

Fundamental in everyday life is the ability to stop or cancel an inappropriate response when this can lead to an improper outcome. Several factors can influence the correct implementation of inhibitory control functions (Diamond, 2013; Friedman & Miyake, 2004). A critical modulating factor during response inhibition is the presence of distracting and conflicting information affecting stimulus–response (S–R) associations and the study of distractor interference on response inhibition functions has seen extensive investigation in the cognitive field (Chambers et al., 2007; Eggert et al., 2022; Mückschel et al., 2016; Ridderinkhof et al., 1999; Verbruggen et al., 2004; Verbruggen et al., 2005; Verbruggen et al., 2014; Verbruggen et al., 2019). Special attention has been given to the role of distractors or interfering information because the ability to cope with distracting inputs is a frequent complication during goal‐directed behavior (Hannah & Aron, 2021). However, our understanding of how S–R mapping processes modulate response inhibition and the neurophysiological dynamic and the functional anatomical structures involved is still incomplete. A means to study the different processes of inhibitory control (Bari & Robbins, 2013) is to examine response inhibition via experimental tasks in which participants are taught to respond to frequent *go* stimuli but are occasionally presented with stimuli that require them to withhold the prepotent response (i.e., proactive inhibition) or to “cancel” the already initiated response process (i.e., reactive inhibition) (Aron, 2011; Verbruggen et al., 2014). While proactive inhibition can be studied with the Go/Nogo task, reactive inhibition can be studied using the Stop Signal task (Logan et al., 1984; Verbruggen et al., 2019). The Stop Signal task is often designed as a choice reaction task with frequent Go trials by assigning a given stimulus (e.g., letters A and B presented visually) to specific response effectors (e.g., left and right index finger). The task's reactive inhibition component consists of presenting a so‐called “Stop Signal” (often a visual or auditory stimulus) with a relatively low frequency after the presentation of the Go stimuli. Participants are instructed to inhibit their initiated/ongoing response when the stop signal occurs. Thus, this task is designed to investigate the ability of the participants to interrupt their response by estimating the stop signal reaction time (SSRT) and measuring the time necessary to successfully cancel/inhibit the ongoing response (Verbruggen et al., 2019). Shorter SSRTs are associated with better action cancellation performance.

Presenting distracting information during a Stop Signal task increases SSRTs (Chambers et al., 2007; Eggert et al., 2022; Ridderinkhof et al., 1999; Verbruggen et al., 2005; Verbruggen et al., 2014) and this pattern has been found in various experiments applying Stroop stimuli (Ridderinkhof et al., 1999; Verbruggen et al., 2004), Flanker stimuli (Chambers et al., 2007; Verbruggen et al., 2004) and perceptual load manipulations (Verbruggen et al., 2014). However, examining interfering effects during response cancellation presents several challenges due to conceptual problems associated with experimental procedures frequently used to induce interferences (Hommel, 2011). However, one conceptually more stringent investigation approach is to implement inhibitory control measures (or stopping) in Simon tasks (Chmielewski & Beste, 2017; Eggert et al., 2023). Unlike other tasks (e.g., Stroop or Flanker tasks), a Simon task does not confound stimulus and response‐related processes during conflict monitoring (Hommel, 2011). In the Simon task, participants respond (left or right) based on a stimulus (e.g., letter “A” or “B”) shown on one side of the screen. Spatial stimuli enhance performance when the response matches the stimulus location (congruent condition) but impair it when mismatched (incongruent condition). Therefore, the combination of the Simon and Signal Stop task results in four response conditions: congruent and incongruent Go trials and congruent and incongruent Stop trials. Conceptually, the dual‐route model (De Jong et al., 1994; Keye et al., 2013) has proposed that response selection in congruent Simon task trials operates via the direct route, requiring little computations during response selection. In incongruent trials, however, a conflict is induced because processing via the direct route has to be controlled by an indirect route to avoid erroneous responding (Keye et al., 2013), thereby enabling a reconfiguration of S–R mappings. The reconfiguration of S–R mappings has also been stressed by the “Theory of Event Coding (TEC)” on the Simon task (Hommel, 2011). According to TEC, the so‐called event files contain representations of how a given stimulus input is mapped onto a motor output. In contrast to the process in congruent trials, in incongruent trials, the information regarding the relevant stimulus dimension (i.e., letter identity) has to be separated from an irrelevant stimulus dimension (i.e., spatial position), which has a strong impact on an associated motor command. Therefore, the information represented in the event file has to be reconfigured in incongruent Simon task trials. As shown before, incongruency in a Simon task affects stopping performance leading to longer SSRTs in congruent than in incongruent trials (Eggert et al., 2023; Verbruggen et al., 2005). Thus, a necessary reconfiguration of the event file's representational content requires reactive inhibitory control.

Several studies have also examined the neurophysiological underpinnings of binding and reconfiguration of event file coding during response selection (Opitz et al., 2020; Petruo et al., 2016; Takacs et al., 2021; Takacs, Bluschke, et al., 2020; Takacs, Mückschel, et al., 2020; Takacs, Zink, et al., 2020) and response inhibition (Eggert et al., 2023; Gholamipourbarogh et al., 2022; Gholamipourbarogh et al., 2023; Prochnow et al., 2021). An innovative method to precisely capture the temporal profiles of neurophysiological correlates of event file dynamics is to apply temporal signal decomposition (i.e., residue iteration decomposition [RIDE]) to EEG data (Ouyang et al., 2015; Ouyang et al., 2017). RIDE decomposes EEG signals into meaningful clusters of activity with distinct functional relevance (Eggert et al., 2023; Opitz et al., 2020; Petruo et al., 2016; Takacs, Mückschel, et al., 2020; Takacs, Bluschke, et al., 2020). Using RIDE, we recently showed that neural activity in a cluster thought to represent event file reconfiguration processes (i.e., C‐cluster), was modulated by the level of interference during reaction inhibition processes (Eggert et al., 2023). Critically, previous findings showed how aspects of event file processing are temporally coded in distinct neurophysiological signals during reactive inhibition processes (Eggert et al., 2023), but did not investigate the strength and temporal stability of event file representations and their spatial distribution in distinct multiple cortical regions. This remains a critical issue considering that event file dynamics have been conceptualized as network dynamics with concomitant processing in multiple cortical regions (Hommel, 2004), a notion which has been supported by neurophysiological evidence (Eggert et al., 2022; Takacs, Mückschel, et al., 2020; Takacs, Zink, et al., 2020). In particular, it has been shown that event file coding involves specific (spatially independent) aspects of neural activity associated with distinct fronto‐parietal cortex regions (Gholamipourbarogh et al., 2022; Gholamipourbarogh et al., 2023; Prochnow et al., 2022). Given these conceptual considerations, we aim to investigate the spatial neural dynamics during reactive inhibition and to examine how representational dynamics unfold in these spatially distinct neural structures. To achieve this, an innovative combination of independent component analysis (ICA), multivariate pattern analysis (MVPA), and source localization analysis is used. Temporal generalization MVPA is an efficient method to examine the differences in the stability of the representational content depending on task condition (King & Dehaene, 2014). Previous findings have shown that MVPA on EEG data captured how S–R bindings during proactive inhibition are associated with distinct neuropsychological signals reflecting the dynamic activation and deactivation of event file representations (Prochnow et al., 2021). Therefore, applying MVPA on spatially distinct neural activity profiles allows for investigating the multi‐regions processing of event file reconfiguration during response cancellation. An elegant method to achieve this is to combine MVPA with ICA. ICA can be used to obtain the topographical location of independent components (ICs) of activity of specific brain sources (Huster et al., 2015; Huster & Raud, 2018). The combination of these methods will provide information about when and how long the distinct temporal profiles of the representational content are present in isolated ICs (Gholamipourbarogh et al., 2023). Based on previous findings showing that a more pronounced off‐diagonal activity reflects event file reconfiguration (Prochnow et al., 2021; Takacs, Mückschel, et al., 2020), we expect a more pronounced off‐diagonal activity in incongruent stop trials, compared to congruent ones, when event file reconfiguration is necessary for the implementation of the response cancellation. Additionally, we assume that distinct functional neuroanatomical structures are associated with the temporally overlapping representations of event file processes. Source localization analysis is therefore used to investigate the functional neuroanatomical regions associated with representational dynamics in the identified components. Considering that previous research has found that medial and superior fronto‐parietal cortices are associated with event file coding (Beste et al., 2023), we also hypothesize that these regions are associated with the representational dynamics observed in the different ICs. However, especially when it comes to conflict monitoring and response selection functions, not only medial prefrontal but also insular cortex functions have been reported (Droutman et al., 2015; Gogolla, 2017). Thus, it is possible that the role of the insula is of relevance in the current study context.

Finally, as outlined above, there are likely multiple ICs in neural activity reflecting the neural dynamics that support conflict‐modulated response stopping. In an explorative analysis, we investigate whether the identified components differ regarding their relevance to explain behavior or whether all of the identified components are relevant to consider for behavioral performance. Importantly, it is essential to consider that the interrelation between neurophysiology and behavior can be nonlinear. Therefore, we apply logistic regression methods. This technique has been previously used for evaluating the nonlinear dependency between parameters, independent of the type of relationship between them.

## 2 MATERIALS AND METHODS

### 2.1 Participants

An a priori power analysis was conducted to calculate the required sample size. Given the novelty of the task, an estimated small to medium effect size *f* = 0.3 was used for both the behavioral and neurophysiological analysis. This yielded a required sample size of *N* = 26, with α error probability of 5% and a power of 95%. However, to account for possible outliers and EEG recording issues that might occur, a total of *N* = 69 participants took part in this study. The final sample size after exclusions (see details below) consisted of *N* = 53 participants (30 males) aged between 18 and 35 (*M* = 25.5, *SD* = 4.65). SSRT estimates have a higher reliability when the stopping probability is close to .50 and a stopping probability range between 0.25 and 0.75 should be considered for data analysis (Verbruggen et al., 2019). Against this background, four participants were excluded. Previous results showed that when the Stop task is embedded with a Simon task, shorter SSRTs are expected incongruent than in congruent trials (Eggert et al., 2023). Therefore, to establish if the SSRTs in this study would show the expected direction, the difference (incongruent minus congruent) between SSRT in congruent and incongruent Stop trials was calculated for each participant with a cut‐off value of 50 ms. One participant with a difference value larger than 50 ms was excluded. Behavioral data were also separately assessed for each condition for possible outliers using the Tukey method implemented in SPSS. Results showed that four participants were identified as outliers in more than one measurement and therefore excluded from the analyses. Two participants were excluded because they scored above the cutoff value in at least one of the scales measured by the adult self‐report used to assess adult (ages 18–59) psychopathology. To achieve homogeneity in the handedness of the sample, one participant was excluded because they reported being left‐handed. Finally, four participants were excluded due to the poor EEG data quality and the low number of remaining epochs for the stop conditions. All participants in the final sample size were right‐handed and reported no psychiatric or neurological illnesses and normal or corrected‐to‐normal vision. Written informed consent was obtained from all the participants before taking part in the study. The study was approved by the Ethical Committee of the Medical Faculty of TU Dresden. The participants received either monetary compensation or course credits for their participation.

### 2.2 Task

**FIGURE 1:**
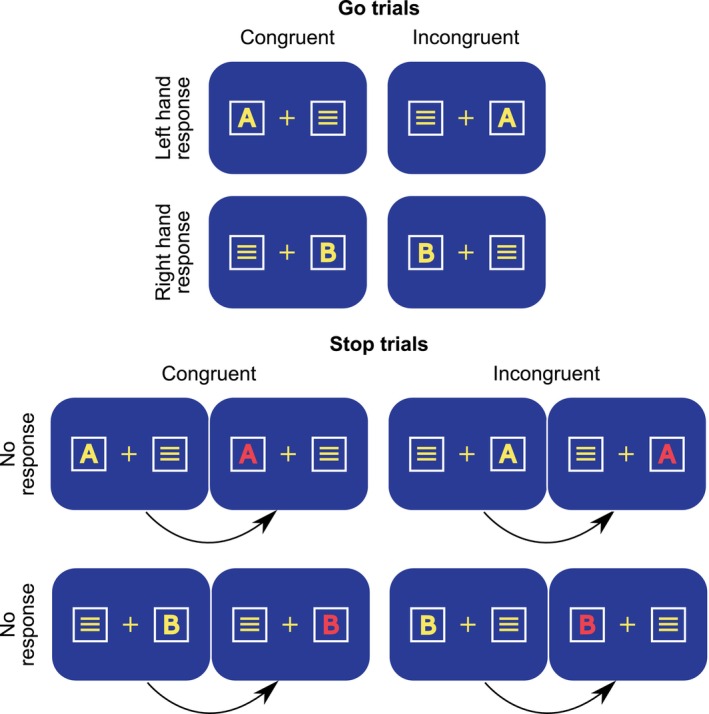
Schematic illustration of the implemented task with all possible stimulus configurations. Congruent and incongruent trials in the Go condition are shown above, while the congruent and incongruent trials in the Stop condition are shown below.

The task used in this study combines the Simon task (Simon & Rudell, 1967; Simon & Small Jr., 1969) and the Stop Signal task (Logan et al., 1984). A schematic illustration of the task is shown in Figure 1. This task has been successfully used to investigate response inhibition and interference processing (Eggert et al., 2023). Participants were comfortably seated in front of a 24″ LCD at an approximate distance of 60 cm. A yellow central fixation cross was displayed at the center of the screen for the entire task duration and was framed horizontally by two white boxes, each 0.7 cm apart, all displayed against a blue background. In each trial, either the letter “A” or the letter “B” was displayed in either the right or the left box. At the same time, a distractor stimulus consisting of three horizontal lines was simultaneously presented in the other frame box. The Simon element of the task was established as follows: the participants were instructed to press the left CTRL button with their left index finger every time the letter “A” was presented and to press the right CTRL button with their right index finger whenever the letter “B” was presented. Notably, this was regardless of the spatial location in which the letter was presented (i.e., left or right box), thus constituting two trial conditions. Whenever the letter “A” was presented in the left box or the letter “B” was presented in the right box, these were coded as congruent trials (i.e., the location of the stimulus corresponded to the side of the responding finger). However, when the letter stimulus was presented on the opposite side of the responding hand, this was coded as an incongruent trial. The participants were instructed to respond regardless of the spatial location of the letter.

The stop signal component of the task was incorporated using Go and Stop trials. Specifically, in Go trials, the letter stimulus was presented either in the left or the right box and lasted for 1700 ms or until a response was given. Go trials with a response could be classified as “correct/hits” or “incorrect.” In the case of no response until 1700 ms, the trial was coded as “miss.” In the Stop trials, the letter stimulus turned red after a variable delay. The stop signal was presented for 1700 ms. In these trials, the participants were instructed to interrupt (i.e., stop) their response. In a stop signal task, it is recommended to achieve a stopping probability of 0.50 (Verbruggen et al., 2019). Against this background, the stop signal delay (SSD) (i.e., the time occurring from the onset of the letter stimulus and the moment it becomes red) was regulated in every stop trial, depending on the prior trial. When a response was correctly withheld after the presentation of the stop signal (i.e., correct rejection), the SSD was increased by 50 ms in the following Stop trial. On the contrary, if the participant failed to withhold the response (i.e., failure to stop), the SSD was decreased by 50 ms in the following Stop trial. The initial SSD was set to 250 ms with a maximum SSD of 1000 ms and a minimum SSD of 50 ms. When reaction times in the previous 50 trials were above 450 ms from the onset of the stimulus, a speed‐up warning “Bitte versuchen Sie, Schneller zu drücken” (German for “please try to respond faster”) was displayed on the screen for 2000 ms. The intertrial interval lasted for 1300 ms. The task consisted of 936 trials, of which 720 trials (77%) were Go trials and 216 (23%) were Stop trials. Furthermore, the Go and Stop trials were equally divided into congruent and incongruent trials. Thus, 360 Go trials were congruent and 360 were incongruent, while 108 Stop trials were congruent and 108 were incongruent. The task was divided into 9 blocks, consisting of 80 Go trials and 24 stop trials. The order of trials was randomized. Before the execution of the experiment, participants were familiarized with the task by performing an exercise version of the task consisting of 36 trials divided into Go and Stop trials. During the exercise task, feedback was provided at every trial regarding the accuracy of the response.

### 2.3 Behavioral data analysis

Repeated‐measures ANOVAs with factors “congruency” (congruent vs. incongruent) and “position” (left vs. right) were used to analyze accuracy and reaction times measures in the Go trials. Repeated‐measures ANOVAs with the same factors were also used to analyze SRRT, error rate accuracy, and error rate reaction times in the Stop trials. Importantly, only correct trials were considered for the reaction times in the Go condition. A Greenhouse–Geisser correction was applied where necessary to take into consideration the potential lack of sphericity. All variables were tested for normal distribution using Kolmogorov–Smirnov tests. In case of significant interaction effects obtained from the repeated measures ANOVAs, these were interpreted using Bonferroni correction statistical hypothesis test. When the assumption of normal distribution was violated, nonparametric Wilcoxon signed‐rank tests were used. Error rate reaction times in Stop trials were compared with reaction times in Go trials using nonparametric Wilcoxon signed‐rank tests to investigate common patterns of results in Stop Signal tasks (Schall et al., 2017). Descriptive statistics are reported using the mean value and the standard error of the mean.

### 2.4 EEG recording and preprocessing

The EEG signal was recorded using a QuickAmp amplifier (Brain Products GmbH, Gilching, Germany) from 60 equidistant Ag‐AgCl‐electrodes with a sampling rate of 500 Hz. The signal was then downsampled to 256 Hz offline. The reference electrode was positioned at Fpz (θ = 90, φ = 90), and the ground electrode was positioned at the coordinates θ = 58, φ = 78. Electrode impedances were kept below 10 kΩ. Offline EEG processing was conducted using the Automagic toolbox (Pedroni et al., 2019) and EEGLAB (Delorme & Makeig, 2004) on Matlab 2019a (The Mathworks Corp). In the first processing steps, flat channels were removed and EEG data were referenced to an average reference. Afterward, the preprocessing PREP pipeline (Bigdely‐Shamlo et al., 2015) and the EEGLAB clean_rawdata() pipeline were applied to the EEG data. The PREP pipeline removes line noise at 50 Hz using a multitaper algorithm and implements an average reference after removing contamination by bad channels. The clean_rawdata() detrends the EEG data by applying an FIR high‐pass filter (>0.5 Hz, order 1286, stop‐band attenuation 80 dB, transition band 0.25–0.75). The Artifact Subspace Reconstruction (ASR, Mullen et al., 2013) was then applied. The ASR is an automatic artifact rejection method in EEG data. Epochs with power over 15 standard deviations relative to the calibration data were reconstructed (burst criterion = 15). Epochs that could not be reconstructed were removed. A low‐pass filter of 40 Hz was also applied (sinc FIR; order = 86; Widmann et al., 2015). EOG artifacts were removed using the subtraction method (Parra et al., 2005). An ICA based on multiple artifacts rejection algorithm (Winkler et al., 2011, 2014) was applied to remove muscle and remaining eye‐related artifacts. ICLabel pipeline (Pion‐Tonachini et al., 2019) was used to identify and remove components containing cardiac artifacts. Removed channels were interpolated using a spherical method.

### 2.5 Signal decomposition (ICA)

The group‐ICA was employed to analyze EEG data and extract subcomponents, as done in previous work (Gholamipourbarogh et al., 2022; Gholamipourbarogh et al., 2023; Yu et al., 2022). The use of group‐ICA was motivated by the fact that traditional ICA is not directly applicable to group data, as it estimates different sets of components for each individual or run, making it challenging to draw inferences about the entire group. An aggregate dataset was created to overcome this limitation by concatenating the pre‐processed data from all subjects. To estimate independent brain components (*C*) from the homogenous neurophysiological activity of EEG data (*X*) across individuals, the group‐ICA approach utilizes the equation *C=WX* (Calhoun et al., 2009). In this equation, *W* refers to the demixing matrix (Hyvärinen & Oja, 2000).

In the first step, the aggregate dataset was subjected to principal component analysis (PCA) to reduce computational complexity. The PCA step retained 98% of the eigenvalues, resulting in a reduced dataset of 20 principal components that captured most of the information. Next, ICA (the fast‐ICA method; Hyvärinen & Oja, 1997) was applied to the reduced and concatenated datasets of all subjects to estimate the ICs of the group data. This step aimed to identify independent patterns of neurophysiological activities within the dataset. The resulting group of ICs provided insights into the underlying brain processes related to the experimental conditions. In the final step, we conducted a back‐reconstruction to obtain individual ICs. Group‐ICA was carried out on data from congruent and incongruent conditions separately, using the EEGIFT toolbox (http://icatb.sourceforge.net/EEGIFT) (Eichele et al., 2011; Rachakonda et al., 2011). Estimated groups of ICs for congruent and incongruent conditions were generated with different orders and scales. So, to facilitate a comparison between the congruent and incongruent conditions, a matching process was performed on the estimated group‐ICs. This matching was achieved using a clustering technique called CORRMAP (http://www.debener.de/) (Viola et al., 2009), which clusters components based on the correlation of their inverse weights. The CORRMAP approach involved initializing each cluster with a group component from the congruent and incongruent conditions and iteratively identifying components with high correlation coefficients (above a specified threshold of 0.85) as homogenous ICs. By applying CORRMAP clustering with 40 different initializations, the study aimed to maximize the identification of similar components for each template. To prevent the clustering process from assigning overlapping components to multiple clusters, a maximum of three represented components from each group (congruent and incongruent) were allowed within each cluster. The group‐ICA was only applied to the Stop trial data.

### 2.6 Multivariate pattern analysis

We employed the MVPA light toolbox to conduct MVPA on the back‐projected matching component pairs obtained from the CORRMAP results (Yu et al., 2022). At the single‐subject level, two analyses were performed: a binary classification to identify time points with different patterns between IC pairs for congruent and incongruent trials and a temporal generalization analysis to examine the temporal dynamics of the representational content at the component ERP level. For the classification analysis, EEG channel amplitudes at individual electrodes were used as features, creating 60 features for the SVM classifier in both conditions. To address the overfitting problem, under‐sampling was applied to balance the number of trials in each class before conducting MVPA for each individual and IC pair. Separate SVM classifiers were trained and validated for each IC pair, using a 5‐fold cross‐validation approach. The classifier was trained on 80% of the data and tested on the remaining 20% in a repeated procedure until all data chunks were tested. Classification accuracy was evaluated using the area under the ROC curve (AUC), and the average performance across test folds was computed as the final performance metric. Wilcoxon tests were performed against chance level (AUC = 0.5) for each time sample across subjects to identify significant time points with reliable classification performance. Cluster‐based permutation tests were applied to correct for multiple comparisons, with cluster‐level statistics calculated based on the sum of Wilcoxon teat values within time points. This permutation process was repeated 1000 times to obtain reliable statistical results.

### 2.7 Source localization

To identify the functional neuroanatomical sources associated with the ICA and MVPA‐detected time windows, the source location method standardized low‐resolution brain electromagnetic tomography (sLORETA) (Pascual‐Marqui, 2002) was applied. Specifically, sLORETA was conducted for the IC pairs in the time windows where MVPA revealed temporal stability. sLORETA provides higher localization precision of deep brain structures compared to other localization methods (Grech et al., 2008), and, most importantly, provides a linear solution to the inverse problem without localization bias (Ocklenburg et al., 2018; Pascual‐Marqui, 2002; Sekihara et al., 2005). Furthermore, previous studies combining EEG/ MRI data and EEG/ brain stimulations have provided evidence for the robustness of sLORETA results (Sekihara et al., 2005). The sLORETA divides the intracerebral volume into 6239 voxels using a spatial resolution of 5 mm within a three‐shell spherical head model. A realistic MNI152 head model (Mazziotta et al., 2001) is used to calculate the standardized current density for each voxel (Fuchs et al., 2002). SLORETA was used to investigate the neurophysiological activation of the congruency effects (congruent minus incongruent trials) in the Stop condition. To do this, the sLORETA built‐in voxel‐wise randomization test with 5000 permutations was used based on statistical nonparametric mapping. Significant differences among voxel locations (*p* < .05) are shown in the MNI brain. Since the current study aims to investigate the interference effect during response inhibition processes (i.e., congruency effect), the sLORETA analysis was conducted only for Stop trials.

### 2.8 Interrelation of neurophysiological and behavioral data

To perform the complementary evaluation of the association between neurophysiological information and behavioral data, we applied a nonlinear regression method using artificial neural network. This technique has been previously utilized for evaluating the nonlinear dependency between parameters, independently of the type of relationship between them. To do this for each specific IC pair, we used a feed‐forward neural network with the input of AUC (averaged across the time window that it was significant) for all subjects. The network's output was the difference in Stop Signal reaction times between the congruent and the incongruent condition. For the cross‐validation, we adopted the leave‐one‐out technique. This entailed treating one subject as the test set while the remaining subjects (*N* = 52) constituted the training set. The neural network was individually trained for each IC pair.

## 3 RESULTS

### 3.1 Behavioral data

#### 3.1.1 Go trials

For the accuracy rate in Go trials, results showed a significant main effect of congruency (*F*_(1,52)_ = 38.445; *p* < .001, *η* ^2^_*p*_ = 0.425) with higher accuracy for congruent trials (97% ± 0.5) than for incongruent trials (94% ± 0.7). Furthermore, a significant interaction of congruency × position was found (*F*_(1,52)_ = 7.337, *p* = .009, *η* ^2^_*p*_ = 0.124). However, when corrected for multiple comparisons, Bonferroni‐corrected Wilcoxon paired *t* tests showed no significant differences between the right and left positions of the stimulus in both congruent and incongruent trials (*all p* > .05). No significant main effect of position was found (*F*_(1,52)_ = 0.025; *p* = .874, *η* ^2^_*p*_ < 0.001). Results for the reaction times in the Go trials showed a significant main effect of congruency (*F*_(1,52)_ = 115.309; *p* < .001, *η* ^2^_*p*_ = 0.689) with faster reaction times for congruent trials (493.7 ms ± 15.56) than for incongruent trials (521.57 ms ± 15.07). Furthermore, a significant main effect of position was also shown (*F*_(1,52)_ = 9.311; *p* = .004, *η* ^2^_*p*_ = 0.152) with faster reaction times for the right (503.04 ms ± 15.45) than for the left side of stimulus presentation (512 ms ± 15.21). However, no significant interaction of congruency × position was found (*F*_(1,52)_ = 0.598; *p* = .443, *η* ^2^_*p*_ = 0.011).

#### 3.1.2 Stop trials

**FIGURE 2:**
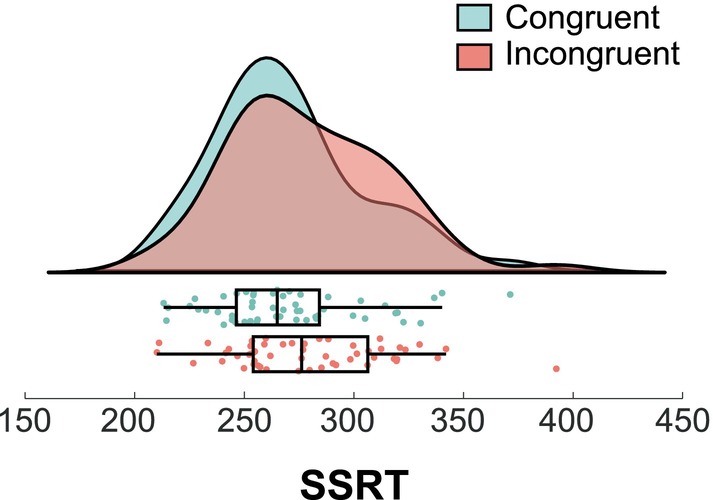
Stop signal reaction time (SSRT) data for congruent and incongruent Stop trials. For each condition, a boxplot, and the individual data points as well as the probability distribution are given.

Results for Stop trials in the congruent and incongruent conditions are illustrated in Figure 2. For Stop trials, there was an error rate (i.e., probability of responding in a Stop trial) of 50.56% (±2.9). The mean SSD was 233.19 ms (±14.69). Furthermore, consistent with the common pattern of results in Stop Signal tasks (Schall et al., 2017), participants had faster reaction times (*z* = −6.334, *p* < .001) in their failures to stop in Stop trials (449.47 ms ±12.05) than in their correct responses in Go trials (567.81 ms ± 30.21). The SSRT was calculated based on the mean estimation method. This method estimates SSRT by subtracting the mean reaction times of go trials with the mean of the inhibition functions, which is the SSD corresponding to the probability of responding equal to 0.5 (Verbruggen & Logan, 2009). For the SSRT, results showed a significant main effect of congruency (*F*_(1,52)_ = 11.999; *p* = .001, *η* ^2^_*p*_ = 0.187) with a shorter SSRT in congruent (269.86 ms ± 4.72) than in incongruent trials (279.01 ms ± 4.89). No significant main effect of position (*F*_(1,52)_ = 0.416; *p* = .552, *η* ^2^_*p*_ = 0.008) nor an interaction of congruency × position (*F*_(1,52)_ = 0.405; *p* = .527, *η* ^2^_*p*_ = 0.008) was found.

For the error rate (i.e., failure to stop during Stop trials) in the Stop trials, the results showed a main effect of congruency (*F*_(1,52)_ = 23.165; *p* < .001, *η* ^2^_*p*_ = 0.308), with a higher error rate for congruent trials (51% ± 0.5) than for incongruent trials (50% ± 0.4). Furthermore, there was a significant interaction of congruency × position (*F*_(1,52)_ = 14.524; *p* < .001, *η* ^2^_*p*_ = 0.218). Bonferroni‐corrected Wilcoxon signed rank tests showed a significant difference between congruent (51.6% ± 0.5) and incongruent (50% ± 0.4) stop trials both on the right side (*z* = −4.769, *p* = 0.002), but not on the left side of the stimulus presentation (*z* = −1.286, *p* > .05). The main effect of position was not significant (*F*_(1,52)_ = 3.893; *p* = .054, *η* ^2^_*p*_ = 0.070).

For error rate reaction times data, the results showed a significant main effect of congruency (*F*_(1,52)_ = 121.160; *p* < .001, *η* ^2^_*p*_ = 0.7), with faster reaction times for congruent (434.78 ms ± 12.28) than for incongruent trials (464.65 ms ± 11.98). A significant main effect of position was also found (*F*_(1,52)_ = 4.721; *p =* .034, *η* ^2^_*p*_ = 0.083), with faster reaction times for the right (445.95 ms ± 12.07) than for left (453.48 ms ± 12.29) side of the stimulus presentation. No significant interaction of congruency × position was found (*F*_(1,52)_ = 0.009; *p* = .93, *η* ^2^_*p*_ < 0.001).

### 3.2 Neurophysiological data

**FIGURE 3:**
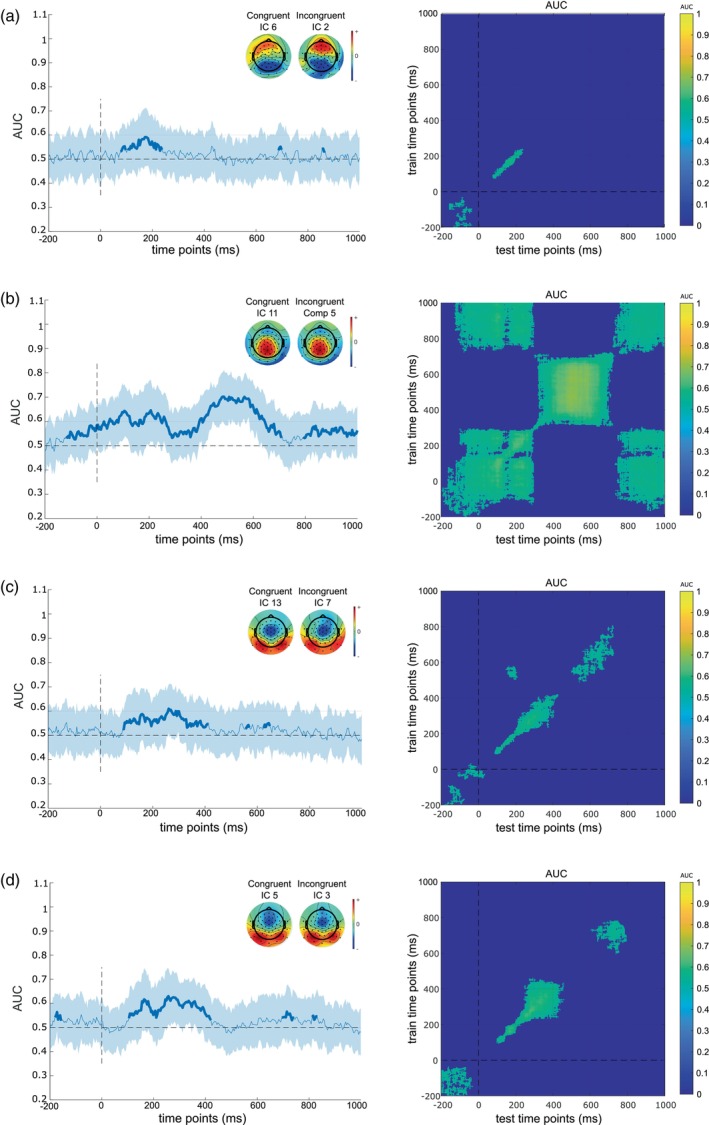
CORRMAP and multi‐variate pattern analysis (MVPA) results for selected independent component (IC) pairs. (a–d) The binary classification performances, the scalp topographies of the selected of the congruent and incongruent conditions (left), and the temporal generalizations (right) of the IC pairs 1–4, respectively. For the binary classification, the shaded error bars represent standard deviation. The scalp topographies reveal the weighting matrices of each IC.

Figure 3 illustrates CORRMAP and MVPA results. The group‐ICA was used to estimate and aggregate back‐reconstructed subcomponents of congruent and incongruent Stop trials in a time window between −200 and 1000 ms. To identify spatially similar components between the congruent and incongruent stop conditions, we implemented the CORRMAP clustering method. This was necessary due to the potential variations in scales and orders of components across different conditions, requiring a matching process between conditions. CORRMAP aggregated components with the same weight distribution on the channels space into a single cluster when a minimum correlation of 85% between weight matrices was found. This process resulted in four pairs of homogenous ICs between congruent and incongruent Stop trials. The ICs' number and the correlation values between the IC topographies of each pair are reported in Table 1. ERP images (scalp topography, trial activity, and ERP signal) for congruent (left) and incongruent (right) conditions are shown in the *supplementary material*.

**TABLE 1:** Similar components for the S‐cluster in the congruent and incongruent NoGo conditions according to the CORRMAP analysis. The correlation coefficient shows the similarity between scalp topographies of each IC pair.

New component number	Original IC number for congruent trials	Original IC number for incongruent trials	Correlation between scalp topographies
1	6	2	0.9
2	11	5	0.99
3	13	7	0.97
4	5	3	0.99

After identifying IC pairs, the back‐projected data at the ERP level were used to investigate the signal‐to‐noise ratio of matching components and to compare them between congruent and incongruent Stop trials. MVPA analysis was applied to each extracted homogeneous back‐projected IC pair to do this. Figure 3 illustrates the respective ICs' topographies, binary classification performance, and the temporal generalization matrix between congruent and incongruent trials. Binary classification performance was significant (i.e. *p* < .05) when AUC > 0.5.

For IC pair 1, the binary classification was significant between 85 and 245 ms, with peaks of 59% between 180 and 187 ms. In IC pair 1, a temporal generalization cluster in the same time interval of the binary classification is also shown. The back‐projected ERP data showed a negative amplitude in both the congruent and the incongruent conditions. For IC pair 2, the binary classification performance was significant (i.e., AUC > 0.5, *p* < .05) between −100 before and 1000 ms after the stimulus onset, with the highest performance of up to 70% at 575 ms. The temporal generalization for IC pair 2 shows a reactivated‐like pattern (King & Dehaene, 2014). In particular, it is possible to observe a sequence of distinct brain activity profiles that reactivated later in time, demonstrating off‐diagonal transient generalization. The activity of the back‐projected ERP showed a negative amplitude between 0 and 300 ms as well as between 700 and 1000 ms and a positive amplitude between 300 and 700 ms in both the congruent and incongruent conditions. For IC pair 3, binary classification performance was significantly higher than the chance level between 90 and 420 ms, with the highest performance of up to 61% between 270 and 275 ms. The temporal generalization cluster centralized around 300 ms, comprising a time period between 100 and 450 ms. The activity at the ERP level showed a positive amplitude between 200 and 500 ms. Finally, IC pair 4 showed significant performance in the AUC between 110 and 430 ms, with the highest performance of 63% at 265 and 285 ms. The temporal generalization showed a clustering occurring mainly in the 250–400 ms time window. The back‐projected ERP data show a positive amplitude in the same time window in congruent and incongruent conditions.

**FIGURE 4:**
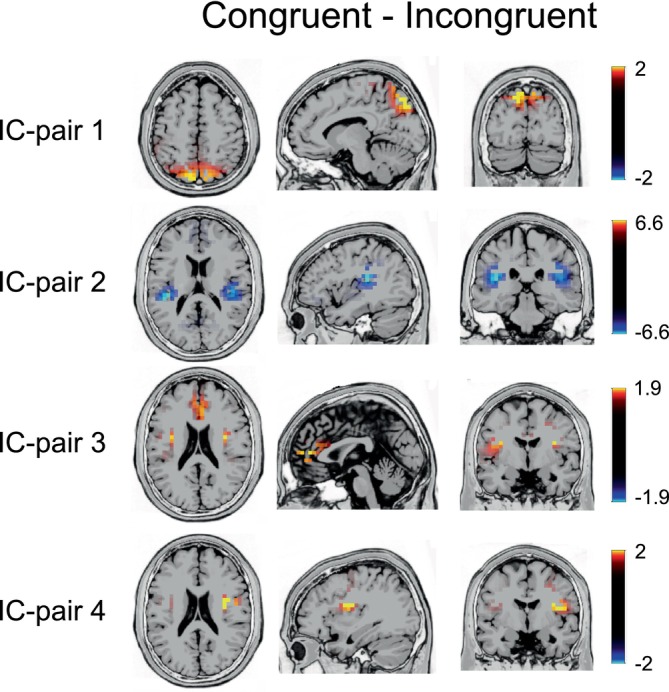
Standardized low‐resolution brain electromagnetic tomography (sLORETA)‐derived maps for selected independent component (IC) pairs (1–4) indicate the sources of maximal differences between congruent and incongruent Nogo trials in time windows showing above‐chance level classification performance and temporal stability.

Source localization results contrasting congruent and incongruent Stop trials are shown in Figure 4. For IC pair 1, sLORETA results showed greater bilateral activation in congruent than in incongruent trials in the superior parietal lobe and the precuneus (BA 7). In IC pair 2, source localization results showed greater bilateral activation for incongruent than for congruent trials in the insula (BA 13) and the superior temporal gyrus (BA 41). For IC pair 3, sLORETA results showed greater bilateral activation for congruent than for incongruent Stop trials in the insula (BA 13), the anterior cingulate (32), and the medial frontal gyrus (10). Finally, in IC pair 4, significantly greater activation in congruent than in incongruent Stop trials was shown in the right insula (BA13) and the precentral gyrus (BA 43).

**FIGURE 5:**
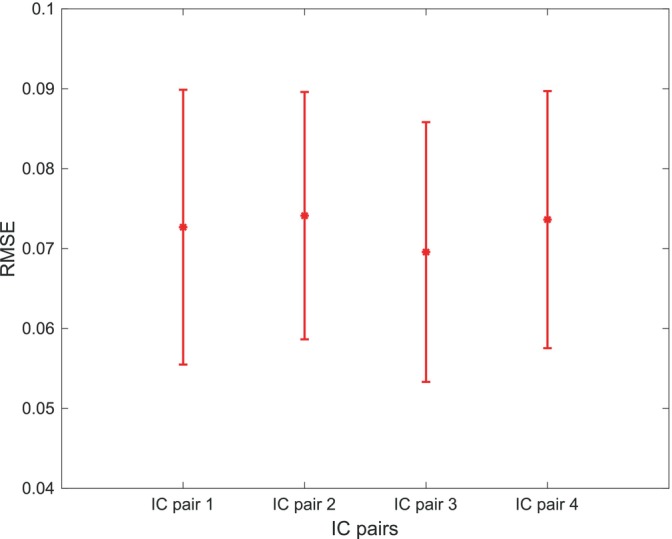
Root mean square error (RMSE) values (with 95% confidence interval) of the nonlinear regression for all independent component (IC) pairs used to find the association between area under the curve (AUC) values and the difference between stop signal reaction times in two congruent and incongruent conditions. Positive values indicate larger activation in congruent Nogo trials, while negative values indicate larger activation for incongruent Nogo trials.

We applied machine learning nonlinear regression methods to perform the complementary evaluation of the association between neurophysiological information and behavioral data. For the cross‐validation, we adopted the leave‐one‐out technique (see Section 2). The root mean square error (RMSE) value for every pair of ICs was used to evaluate the accuracy of predicting behavioral data using the mean AUCs. The mean RMSE values (with a confidence interval of 95%) for each IC pair are depicted in Figure 5. The small values of the prediction errors show that the ACU values were able to predict behavioral data efficiently. The statistical comparison (*t* test) between the error values did not show any significant difference (*all p* > .473), implying that none of the IC pairs had a greater impact on the behavioral outcomes than the others and that all the components had the same influence.

## 4 DISCUSSION

Coping with distracting inputs during goal‐directed behavior is a common challenge, especially when halting behavior or responses that are no longer necessary or have become inappropriate. The neural basis for this remains debated. Our study explores this using a conflict‐modulation Stop Signal task, integrating group‐ICA, MVPA, and EEG source localization analysis.

### 4.1 Behavioral findings

At the behavioral level, results replicated findings showing that stopping performance (i.e., SSRTs) was better in congruent than in incongruent trials (Eggert et al., 2023) using the same combination of a Simon and a Stop Signal task, and corroborated other studies showing that interfering information has a detrimental effect on response cancellation performance (Chambers et al., 2007; Ridderinkhof et al., 1999; Verbruggen et al., 2014). It is possible to explain the better stopping performance in congruent than incongruent trials when considering the dual route model of the Simon task (Keye et al., 2013). In congruent trials, the corresponding S–R association is resolved by simple automatic processes operated via the direct route in both the Go and Stop trials. In incongruent trials, response processes have a higher level of complexity due to the conflict between the activation of both the automatic direct route and the conditional indirect route. The increase in complexity increases SSRTs and leads to reduced stopping performance. The effects of interfering information on S–R mapping reconfiguration during stopping performance in incongruent trials can be further explained through the TEC framework (Hommel et al., 2001), which also accounts for Simon‐task S–R conflicts (Hommel, 2011): Interference effects in incongruent Simon trials emerge because the event file specifies that the mappings of stimulus features to response features need to be reconfigured to respond appropriately. This time‐consuming process (Hommel, 2009) interferes with the process of response stopping, as evidenced by longer SSRTs in incongruent than congruent trials. The important question answered by the neurophysiological data analysis is how the representational content of event file dynamics underlying this (replicable) behavioral effect pattern is processed at a neural level. We also reported the results for the error rate probability for completeness. Results showed a higher error rate probability in congruent compared to incongruent stop trials, with a 1% error rate probability difference between conditions. The current finding might have occurred due to spurious differences rather than a meaningful effect of congruency. Similarly, previous studies combining the Simon and Stop‐Signal task have not formulated hypothesis on the effect of congruency on error rate (Eggert et al., 2023; Verbruggen et al., 2005). This is because in the experimental design, the signal stop delay is constantly adjusted with a staircase tracking procedure in order to obtain a stopping probability of 50%, separately for congruent and incongruent trials (see Section 2). For this reason, it would be speculative to draw any conclusion from this finding.

### 4.2 Neurophysiological findings

The neurophysiological data show that there are four spatially independent neural activity profiles (cf. IC analysis) showing modulations between congruent and incongruent Stop trials. This already suggests that different neural subprocesses subserve the dynamics of the reconfiguration of the event file representations. Of note, the results of the neural network analysis interrelating neurophysiological and behavioral data show that each of the identified components is of similar importance in predicting behavioral modulations between the contrasted experimental conditions (i.e., stopping in congruent and incongruent trials). Previous findings on conflict‐modulated proactive inhibitory control processes also suggest that multiple independent spatial activity profiles underlie event file reconfiguration (Gholamipourbarogh et al., 2022; Gholamipourbarogh et al., 2023) and the same has been found for response selection (Takacs, Mückschel, et al., 2020). Crucially, the event file concept assumes distributed processing of perceptual and motor aspects during goal‐directed action control (Hommel, 2004). The current findings corroborate an emerging pattern according to which different independent neural activity patterns mediate perception‐action integration during various instances of response selection and control. Intriguingly, the source localization analysis for the current data suggests that the representational dynamics reflected by spatially independent neural activity profiles, especially the insular cortex (BA13) play an important role in reactive inhibitory control processes. The reason is that in three out of four ICs (i.e., except IC‐pair 1), the insular cortex reflected activity modulations alongside other cortical regions.

IC‐pair 1 shows a significant binary classification performance between 85 and 245 ms after stimulus presentation, with limited off‐diagonal decoding (i.e., temporal generalization) (King & Dehaene, 2014). For this IC‐pair, source localization results revealed greater activation in the congruent than incongruent stop condition in the precuneus (BA 7). Evidence has demonstrated the role of the precuneus and parieto‐occipital regions in attention processes, which are relevant in Simon task S–R conflicts (Ghin et al., 2022; Leuthold, 2011; Vahid et al., 2020; Wascher et al., 2001; Wiegand & Wascher, 2005). Previous findings have suggested that ERP positivity around 200 ms (i.e., P2) during a Simon task might reflect the early allocation of attentional resources (Ghin et al., 2022). The regions of the precuneus and parieto‐occipital regions (BA7) contribute to the selection of motor responses (Bernier et al., 2012; Cisek & Kalaska, 2002; Jaffard et al., 2008; Sulpizio et al., 2017), possibly because the superior parietal cortex plays a central role in S–R translation processes (Gottlieb, 2007). From that perspective, it seems that IC‐pair 1 reflects the initial representations necessary to perform S–R translation processes, which are then followed up by processes reflected in IC‐pairs 2, 3, and 4. This interpretation is substantiated by the finding that all IC‐pairs explained behavioral performance to a similar extent. Such a result is unlikely to occur if the representations reflected by the different IC‐pair were substantially different. Thus, the findings suggest that the representations processed are highly similar despite different associated spatial neural activity patterns and the functional neuroanatomical structures associated. Importantly, differences in the IC‐pairs and associated functional neuroanatomical structures seem to be a function of the time that has elapsed after presenting the target stimulus which determines whether a response had to be stopped. This is reflected by the fact that for all of the other IC‐pairs significant time windows of MVPA decoding extended beyond the time period shown for IC‐pair 1.

The decoding of representations in IC‐pair 2 was associated with the insular cortex (BA13). The insula cortex and adjacent regions in the superior temporal gyrus have been previously associated with response‐updating functions during decision‐making, and neuroimaging studies have demonstrated the involvement of the insula cortex in a plethora of functions (Droutman et al., 2015; Gogolla, 2017; Uddin et al., 2017), from sensory functions, automaticity and motor control, decision‐making, social functions, and self‐awareness (Gogolla, 2017). Evidence shows the insula cortex's involvement in conflict monitoring and error detection functions during Simon task performance (Ham et al., 2013; Rosenberg et al., 2021; Son et al., 2023). Most importantly, the MVPA showed significant decoding of the representational content and off‐diagonal activity in the entire examined trial period and the temporal generalization matrix (cf. Figure 3) revealed a checker‐board‐like pattern, which suggests that representations become reactivated periodically (King & Dehaene, 2014). This pattern has to be seen in the context of the interpretation of IC‐pair 1. The decoding of representational content in IC‐pair 2 was evident in periods before this was possible in IC‐pair 1. Thus, relevant representational content is processed by insular cortex structures and superior parietal structures early on. However, representational content associated with the insular cortex (BA13) was also decodable in periods after the time window where decoding was possible for IC‐pair 2. The representation reactivation pattern observed for IC‐pair 2 may reflect reiterant processing of representational content relevant for early attentional selection stages (IC‐pair 1) and processes relevant for action cancellation (stopping) in complicated (congruent) and non‐complicated (incongruent) S–R constellations. Activation differences between congruent and incongruent trials in the insula cortex were also evident in IC‐pairs 3 and 4. However, unlike IC‐pair 2, there was a greater activation for congruent than incongruent Stop trials. Even though IC‐pairs 3 and 4 share some similarities with regard to the diagonal decoding and temporal generalization profile, distinct neural activation patterns can still be observed in the AUC performance and source localization results. In particular, IC‐pair 3 was associated with higher activation in congruent than incongruent trials in both the right and left side insula (BA13), the anterior cingulate (BA32), and the medial frontal gyrus (BA10). In contrast, the IC‐pair 4 was associated with higher activation in congruent than incongruent trials in the right insula (BA13) and the precentral gyrus (BA4). The opposite activation patterns in the insula region between congruent and incongruent trials and different temporal generalization patterns observed in the IC‐pair 2 compared to IC‐pairs 3 and 4 could be explained by the dual route model of the Simon task (De Jong et al., 1994). As described above, the direct (automatic) route is mainly involved during congruent trials, whereas incongruent trials involve both direct and indirect routes. The reactivation of representational content relative to the IC‐pair 2, as illustrated by the temporal generalization profile, indicates the occurrence of more complex processes. This suggests that the isolated IC‐pair captures the response selection and stopping processes mediated by the indirect route, in line with the greater activation in the insula cortex for incongruent than congruent trails. On the other hand, the limited off‐diagonal temporal generalizations in the IC‐pairs 3 and 4 indicate relatively automatic processing (i.e., congruent S–R mapping) mediated by the direct route. This also would explain the greater activation in the congruent condition than in the incongruent trials in the insula cortex. A unifying explanation of these results suggests that the insula cortex is critical for both routes and is involved in distinct cognitive subprocesses during response cancellation. This aligns with the suggestion that the insula cortex functions as a brain hub, relaying neural information from and toward different neural systems (Gholamipourbarogh et al., 2022; Gogolla, 2017). The TEC framework could also provide a complementary explanation of these results. According to TEC, when incongruent trials occur, the operating event files need to be reconfigured to account for the discrepancy between irrelevant stimulus location and the triggered prepotent response. As current behavioral and prior neurophysiological evidence suggests (Eggert et al., 2023), the reconfiguration of the event file requires a longer processing time when the ongoing response must be canceled. Thus, the temporal generalization of the representational content in the IC‐pair 2 might reflect the reconfiguration of the event files. In line with this assumption, previous studies have shown that longer off‐diagonal temporal generalization can be observed in experimental conditions requiring more complex cognitive operations compared to less complex conditions (Eggert et al., 2022; Prochnow et al., 2021).

### 4.3 Conclusions

In summary, we show that stopping performance (SSRTs) is better in congruent (nonconflicting) trials than in incongruent (conflicting) trials, consistent with previous findings. Conflict effects in incongruent trials compromise stopping more due to the need of reconfiguration of S–R mappings. Of note, these cognitive dynamics are reflected by four independent neural activity patterns (ICA analysis) each coding representational content (MVPA). It is shown that each component was equally important in predicting behavioral outcomes. The data support an emerging idea that perception‐action integration in action‐stopping involves multiple independent neural activity patterns. One pattern relates to the precuneus (BA 7) and is involved in attention and early S–R processes. Of note, three other independent neural activity patterns were associated with the insular cortex (BA13) in distinct time windows. These patterns reflect a role in early attentional selection but also show the reiterated processing of representational content relevant for stopping in different S–R mapping contexts. Moreover, the insular cortex's role in automatic versus complex response selection in relation to stopping processes is shown. Overall, the insular cortex is depicted as a brain hub, crucial for response selection and cancellation across both straightforward (automatic) and complex (conditional) S–R mappings, providing a neural basis for general cognitive accounts on action control.

## AUTHOR CONTRIBUTIONS

All authors had full access to the data, gave final approval for publication, and agree to be held accountable for the work performed therein. *Conceptualization*: F.G., E.E., and C.B. *Software*: N.G. and N.T. *Investigation*: E.E. *Formal analysis*: F.G., N.G., and N.T. *Writing*—*original draft*: F.G., E.G., N.G., and C.B. *Writing—reviewing and editing*: C.B. *Visualization*: F.G. and N.G. *Supervision*: C.B. *Funding acquisition*: F.G.

## FUNDING INFORMATION

This work was supported by a grant from the Else Kröner Fresenius Foundation to F.G. (2022_EKEA.188).

## CONFLICT OF INTEREST STATEMENT

The authors declare no conflicts of interest.

## Supporting information


**DATA S1** Supplementary Information.

## Data Availability

The data that support the findings of this study are available from the corresponding author upon reasonable request.
