# Supplementary material for: Response stopping under conflict: The integrative role of representational dynamics associated with the insular cortex
Source: Hum Brain Mapp. 2024 Apr 25;45(6):e26643. doi: 10.1002/hbm.26643 (PMC11046082; doi:10.1002/hbm.26643)
Supplement: Supplementary file 1 — DATA S1 Supplementary Information. [file HBM-45-e26643-s001.docx]

Supplemental Material

**Response Stopping under Conflict: The Integrative Role of Representational Dynamics associated with the Insular Cortex**

Filippo Ghin, Elena Eggert, Negin Gholamipourbarogh, Nasibeh Talebi, Christian Beste^#^


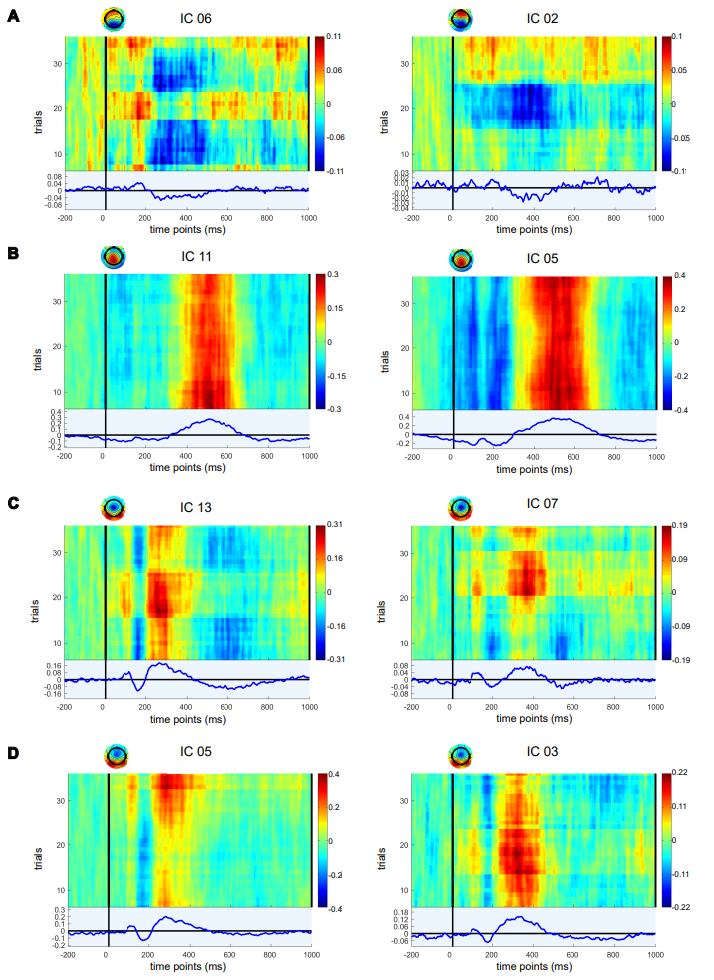


Figure S1. (A - D) illustrates the ERP image (scalp topography, trial activity and ERP signal) of the selected IC pairs 1 to 4 for two congruent (left) and incongruent (right) stop conditions in the time between -200 to 1000 ms respectively. Color bars show trial activities.
